# Artisans and dugout canoes reveal pieces of Atlantic Forest history

**DOI:** 10.1371/journal.pone.0219100

**Published:** 2019-06-26

**Authors:** Laís Lima de Paula, Michele Dechoum, Viviane Stern Fonseca-Kruel, Neusa Tamaio, Natalia Hanazaki

**Affiliations:** 1 Programa de Pós-Graduação em Biologia de Fungos, Algas e Plantas, Universidade Federal de Santa Catarina, Florianópolis, Santa Catarina, Brazil; 2 Departamento de Ecologia e Zoologia, Programa de Pós-Graduação em Ecologia, Universidade Federal de Santa Catarina, Florianópolis, Santa Catarina, Brazil; 3 Instituto Jardim Botânico do Rio de Janeiro, Rio de Janeiro, Brazil; Missouri Botanical Garden, UNITED STATES

## Abstract

Dugout canoes are boats made from a single tree trunk. Even with the modernization of fishing, they are still made and used for artisanal fishing on the coast of southern and southeastern Brazil and in other regions of the world. Various tree species are used to construct these canoes and choosing a species is related to characteristics of the location, available raw materials and purpose of the boat. Our objective was to better understand the variation in dugout canoes in relation to tree species, tree size and fishing use, over time, along a coastal strip of southern and southeastern Brazil within the Atlantic Forest domain. We interviewed 53 artisans and analyzed 358 canoes that ranged from 1 to around 200 years old. *Schizolobium parahyba* is currently used the most. In the past, species of the family Lauraceae (*Nectandra* sp. / *Ocotea* sp.) were frequently used, as well as *Cedrela fissilis* and *Ficus* sp. The size of the canoes varied based on time, coastal region, environment where the boat is used (exposed or sheltered) and type of fishing. The average size of recent canoes was smaller than older canoes for more common species (*S*. *parahyba* and *C*. *fissilis*), reflecting changes in the vegetation of the biome over time, both in the species and size of individuals available. Latitudinal variation can also influence the availability of tree species along the studied regions. An increase in environmental monitoring has contributed to a decline in constructing dugout canoes, resulting in the use of fiberglass canoes and other motorized boats. Although canoe size varied based on region, location and use, today some of the older canoes represent large trees of the past and pieces of Atlantic Forest history.

## Introduction

Dugout canoes were among the first types of boats constructed and used by humans on practically all continents [1–4]. They are called dugout canoes because they are sculpted from a single tree trunk [5]. The historical use of this type of boat in different parts of the world is reported in dendrological studies that note canoes have existed for centuries or millennia, such as those encountered in New Brunswick (Canada), from 1557 [3], and Slovenia, from 3160 to 3100 B.C. [2]. In Japan, more than 100 dugout canoes dated to the Holocene period (postglacial) were discovered, mostly from Lake Biwa, which were used for fishing and transportation [4].

In Brazil, the technique for making canoes was originally developed by indigenous peoples and later modified by European immigrants [6]. During colonization, Europeans initially occupied coastal regions of the country. These communities mainly practiced agriculture and fishing for consumption and constructing boats was necessary for these activities [7]. At this time, Brazil became a support point for boat repair and supply because many regions were rich in wood, especially coastal forests [8, 9]. Dugout canoes underwent many transformations and adaptations over time due to the physical characteristics of each location, such as the amount of wind, ocean currents, depth of the ocean and rivers, availability of raw materials (plant resources), boat use [10] and type of fishing [11].

Although there are still dugout canoes in Brazil and other countries, knowledge about the use of tree species in the construction of traditional boats is in the process of being forgotten [12, 13]. In nearly all regions of Brazil, traditional canoes are being replaced by aluminum or fiberglass boats, resulting in the loss of hundreds of years of traditions and knowledge synthesized in each traditional boat [14]. In Micronesia, a decline in traditional knowledge about boat building was observed [13]; the ability to make canoes is on its way to extinction since younger people reported they lack knowledge about canoe building, which is especially worrying in societies that depend on these boats [15].

On the coast of Santa Catarina State, Brazil, the 1960s were recognized by artisans (canoe builders) as the start of the difficulties related to the extraction of raw material to construct dugout canoes. This is especially due a major increase in environmental monitoring, modernization and incentive for technological development during this period, which contributed to a decline in the practice [16]. In parallel, changes in the vegetation cover of the Atlantic Forest domain resulted in a loss of habitat and fragmentation [17,18], which could have altered the availability of plant resources that had traditional uses, contributing to changes in the habits of human populations in coastal regions. Presently, areas of dense ombrophilous forest that lack traces of past use are rare, reflecting the long history of transformation that resulted from different groups of humans that interacted and interact with the environment [18].

Some isolated studies have investigated dugout canoe building on the Atlantic coast of Brazil [11,16, 19], but there are no previous studies about this subject that evaluate variations in the use of tree species over time (especially the last four decades) and between regions. This study, using an ethnobotanical approach, sought to understand variations in the use of tree species from a temporal and spatial perspective. We tested the following hypotheses: 1) the tree species used to construct canoes varies over time and between the southern and southeastern regions; and 2) canoes size varies over time, for the type of fishing, and between the southern and southeastern regions. We expected the following: variations in the tree species used to build the canoes in the past and present, reflecting the availability of tree species in the Atlantic Forest over time; variations in the choice of tree species used to build canoes along a latitudinal gradient, depicting phytophysiognomic differences in the Atlantic Forest biome, mainly for dense ombrophilous forest that is common in the study area; and that recently built canoes would be smaller than older canoes, reflecting that larger trees were more available in the past.

## Materials and methods

### Study area

The study area is part of the coast of the Southeast and South regions of Brazil, from Cabo Frio, in the state of Rio de Janeiro, to Cabo de Santa Marta, in the state of Santa Catarina. This area corresponds to the coast of the crystalline escarpments, according to the compartmentalization of the Brazilian coast that follows oceanographic, climatic and geomorphological parameters [20]. The landscape is very diverse in these coastal regions, including more exposed environments with different types of beaches and more sheltered environments with estuarine complexes formed by canals, rivers, bays and coves [20, 21, 22]. All of the study area is within the Atlantic Forest, one of the world’s biodiversity hotspots with high levels of endemic and threatened species [23]. The vegetation is mostly composed of subtropical evergreen rainforest [24], characterized by large- and medium-sized trees, lianas and abundant epiphytes [25], with different compositions of plant species along a latitudinal gradient [26].

**Fig 1:**
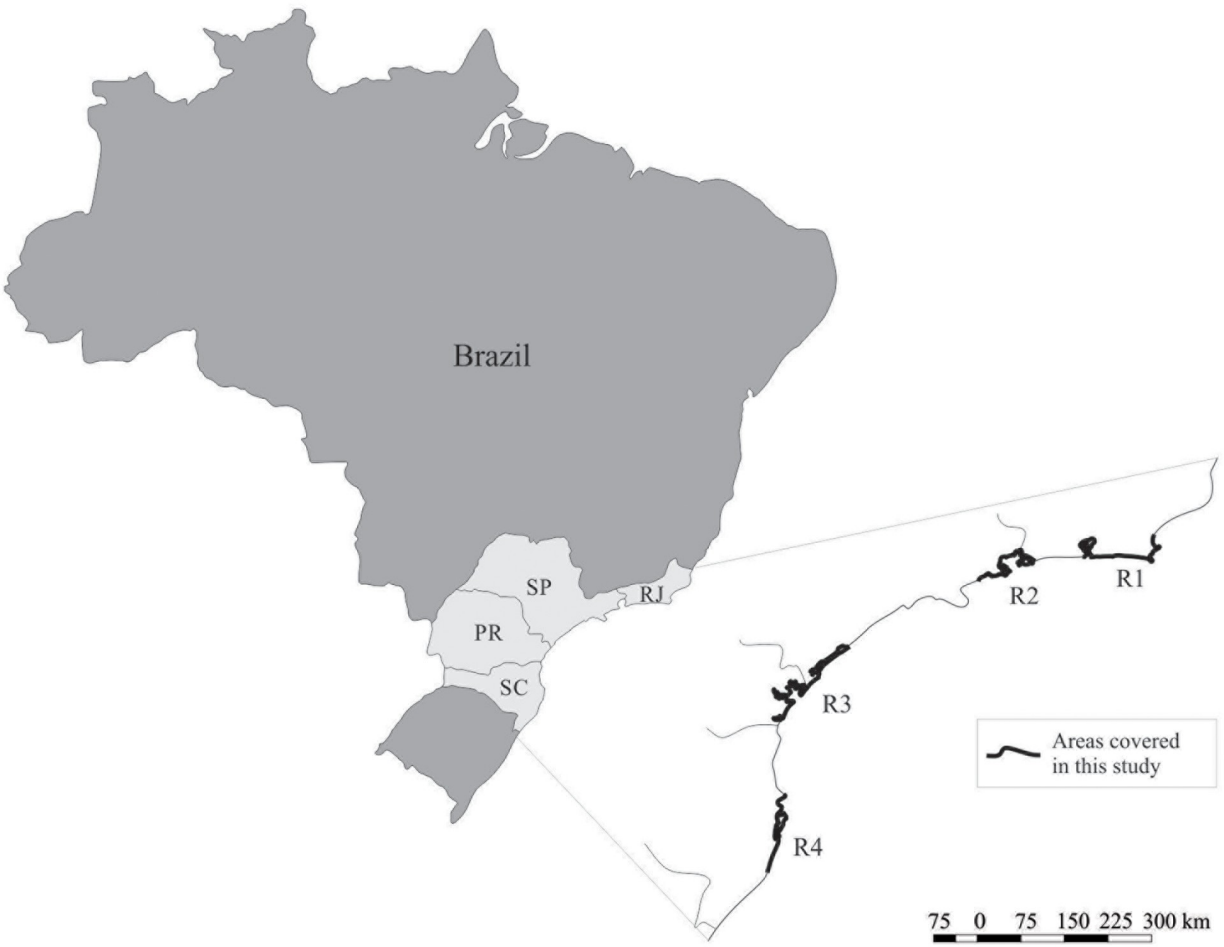
Study area and four regions (R1, R2, R3 and R4).

We divided the study area, with a latitudinal range of 22°S to 28°S, into four regions (Fig 1; S1 Table): furthest north, region 1 (R1) includes the region of Cabo Frio to the city of Rio de Janeiro; followed by region 2 (R2), which includes the southern coast of the state of Rio de Janeiro and the northern coast of the state of São Paulo (Paraty to Ubatuba), with shorter and straighter beaches and no extensive coastal plains; region 3 (R3), which includes the southern coast of the state of São Paulo and the state of Paraná, or Lagamar, with extensive coastal plains that separate the coastline from the plateau [21] and formations of mangrove and sheltered environments; and the southernmost region 4 (R4), which includes the coast of Santa Catarina that has numerous bays, coves and beaches [21].

### Data collection

After obtaining written and oral free informed consent, we collected the data through interviews with artisans, measuring dugout canoes, and collecting wood for identification. We conducted expeditions to collect data between February 2016 and August 2017, after obtaining authorization to collect botanical material (Brazilian Federal Environmental Agency/ICMBio, through Sistema de Autorização e Informação em Biodiversidade/SISBIO 53029–1), conduct research with human beings (Universidade Federal de Santa Catarina Ethics Committee 45797715.2.0000.0121), and access traditional knowledge (National System for Management of Genetic Heritage and Associated Traditional Knowledge/SISGEN AEB5C33).

Sampling was intentional and non-probabilistic [27], based on indications of working or retired artisans with experience in canoe building (key informants). We conducted participant observation and semi-structured interviews after free and informed consent about the activity and canoe building [28]. Additionally, we interviewed canoe owners, people that participate as helpers in the building process and fishers that know about the history of canoes.

The questionnaire used as the basis for the interviews (S1 File) consists of questions about the tree species used to construct canoes (currently and in the past), criteria used to choose the species, aspects about the environment where the canoe is used (e.g., sandy beaches, bays, sheltered estuaries, lagoons) and type of canoe used in relation to type of fishing. When possible, we conducted guided tours to recognize mentioned trees and collect botanical material for identification. We identified the collected specimens by consulting botanical works [29, 30] and specialists. We identified non-collected species using other published works [16, 19, 31–37]. We used the database CNCFLORA [30] to verify the scientific names of the identified tree species.

**Fig 2:**
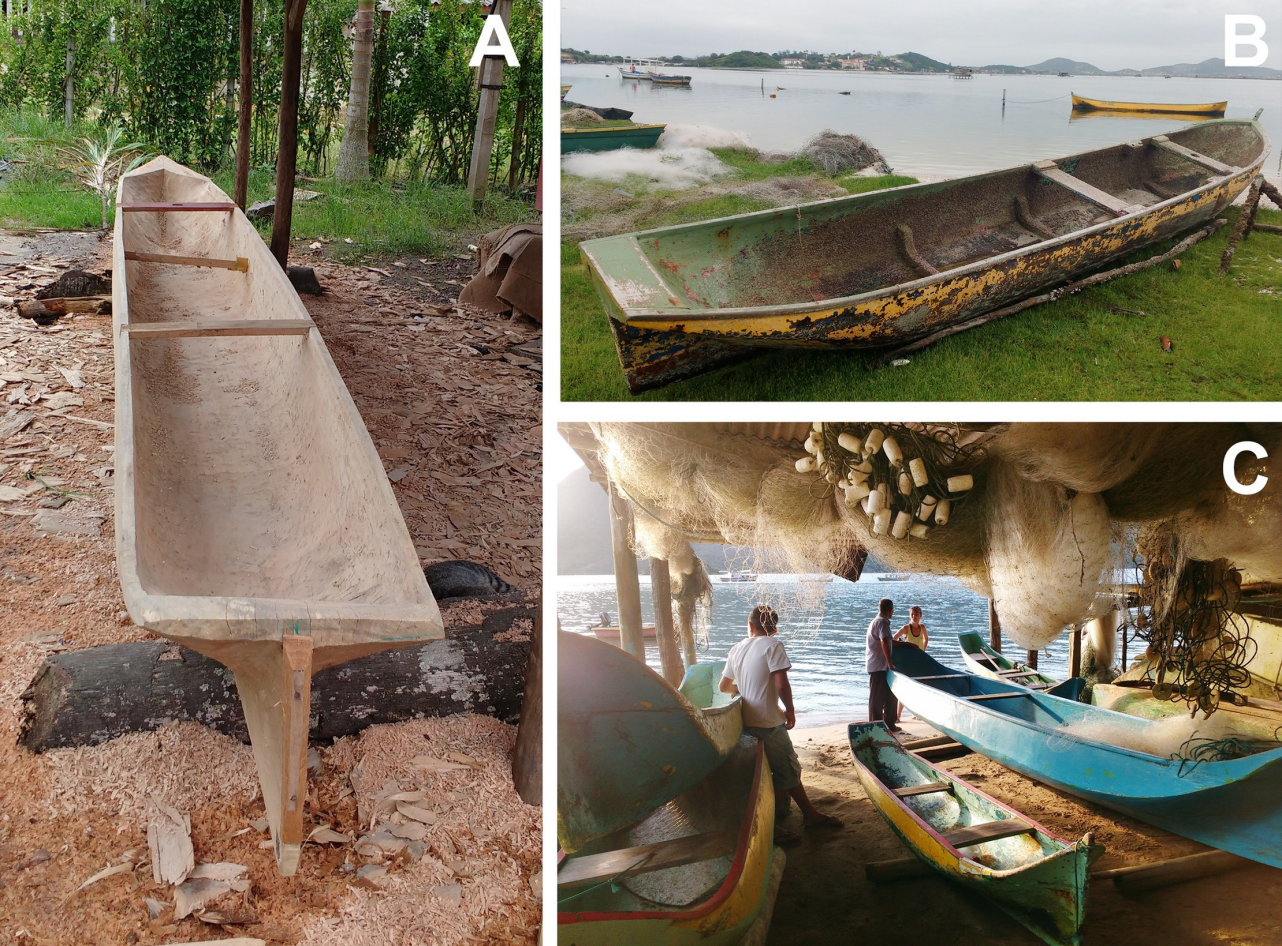
Examples of the canoes sampled. (A) Canoe under construction in Antonina (Paraná State, R3), made with *Vochysia bifalcata*, length 5.9m; (B) Canoe with about 70 years old in São Pedro da Aldeia (Rio de Janeiro State, R1), made with Clarisia racemosa, length 6.44; (C) Several canoes in Saco do Mamanguá (Rio de Janeiro State, R2): large blue canoe with 17 years made of *Tachigali* sp., 7.6m length; small yellow canoe with 16 years made of *T*. *denudata*, 3.13m length.

In each region of the study area we found existing canoes. For each canoe, we noted the following: tree species used; age (year of construction); length and width; environment where it is used; and predominant type of fishing (Fig 2). Data was collected for 221 canoes. We used secondary data from 130 canoes from Ubatuba [19] and seven canoes from Arraial do Cabo [38], totaling 358 canoes analyzed. When possible, we collected a small piece (splinter) of wood of the canoe hull to verify the botanical species through microanatomical analysis. In some cases, the artisan had pieces of the wood used to construct the hull, which was also collected and used for identification.

We sent the wood samples collected in the studied areas to the JBRJ Plant Anatomy Laboratory (JBRJ Laboratório de Anatomia Vegetal) for analysis and to archive in their xylotheque (RBw). To identify the wood/species, each sample was polished with sandpaper and water and photographed using the software Image Manager (IM50) and a Leica camera coupled to a Leica stereomicroscope (model MZ16). We conducted a macroscopic analysis with a 10× linen test magnifier and compared our samples with those in the Rio de Janeiro Botanical Garden Research Institute (Instituto de Pesquisas Jardim Botânico do Rio de Janeiro–RBw) and Botanical Institute (Instituto de Botânica–IBT, São Paulo) xylotheques. The anatomical terminology follows Coradin and Muñiz (1991) [39]. Fertile plant samples were identified by botanical specialists and subsequently deposited in the herbarium FLOR at the Federal University of Santa Catarina (Universidade Federal de Santa Catarina–UFSC). Sterile samples were incorporated in the herbarium EAFM at the Federal Institute of Education, Science and Technology, Campus Manaus-Zona Leste (Instituto Federal de Educação, Ciência e Tecnologia do Amazonas, Campus Manaus-Zona Leste–IFAM-CMZL).

### Data analysis

We divided the canoes into two age groups: recent, representing the present; and old, representing the past. To establish the age limits of the canoes of each group, we used (through the questionnaire cited above) the average of what is the “past” according the artisans, which was 41 years ago. Therefore, canoes more than 41 years old represented the old group and canoes up to 41 years old represented the recent group. When the interviewee did not exactly know the age of the canoe, the age was estimated using temporal references remembered by the interviewee, for example, commemorative dates and historical events. To test the existence of variation in tree species used in the present and in the past, and if there is variation in the tree species for the regions (R1, R2, R3 and R4), we used a PERMANOVA test with 999 permutations for each data group (information obtained from the interviews and examination of canoes) and calculated the Jaccard dissimilarity, where the tree species represented the response variable and the time (old canoes and recent canoes) and the regions represented explanatory variables.

We calculated the relative frequency of the tree species cited by the artisans, for past and present use, and the absolute frequency of the tree species used in the construction of old and recent canoes, in order to verify the variation of species used over time. Additionally, we calculated the absolute frequency of the tree species used in the construction of canoes by region to identify variation in the species used for canoes among the regions.

To verify if canoe size (response variable) varied in function of canoe age (predictor variable), we conducted a linear regression analysis using the data from the 358 canoes (250 recent, up to 41 years old, and 108 old canoes). We conducted a t-test to verify if there were differences in the size of the recent and old canoes for the two most frequent tree species (*C*. *fissilis* = 39; *S*. *parahyba* = 127). To test if variation exists in canoe size for the regions (R1, R2, R3, R4), we conducted an analysis of variance (ANOVA) and Tukey's a posteriori test to compare each pair of regions. The assumptions of normal distribution and homoscedasticity were met so that parametric tests could be used. Since width and length of the canoe are two highly correlated variables (for 358 canoes, r = 0.82; gl = 356; p<0.01), we tested only one of the variables (width) in the analysis involving variation in canoe size.

We tested other explanatory co-variables related to size variation of the canoes, including environment were the canoe is used (sheltered or exposed) and type of fishing. We separated the canoes into two groups: those used in sheltered environments (i.e., bays, estuaries, lagoons, coves or rivers) and exposed environments (open ocean). Due to the non-normality of the data, we used the Wilcoxon test (nonparametric equivalent of the t-test) to compare the measure of the central tendency of the size of the canoes used in the sheltered and exposed environments. Finally, we identified the predominant types of fishing that the canoes are used for to test if canoe size varies in function of fishing type, which was done with the Kruskal Wallis test. We conducted the analyses with the R version 3.3.3 platform and the continuous variable were transformed to log_10_.

## Results

We interviewed fifty-three artisans (R1 = 1, R2 = 9, R3 = 17, R4 = 26). R1 is peculiar because canoes are not built in the region; only finishes and repairs are made to the boats. However, the artisan in R1 was considered a canoe builder since the individual makes large repairs to these boats. In R1, even the oldest canoes were brought pre-excavated from other regions because there were and are still no large trees ideal for this purpose. The interviewees cited 75 names of trees for building dugout canoes. Twenty of these names were cited by only one interviewee, 19 only in the analysis of the canoes and 36 names were cited during both activities. We identified 30 species and 12 genera (Table 1).

**Table 1:** Trees used in the manufacture of dugout canoes according to the interviews and canoes analyzed. Ncit = number of citations (PR = present; PS = Past), Nca = number of canoes.

Family	Local name	Ncit		Nca	Voucher or [bibliography]
*Tree species*		PR	PS		
**Apocynaceae**					
*Aspidosperma* sp.	peroba; perova	0	9	0	[16]
	peroba-rosa	0	1	0	[16]
**Araucariaceae**					
*Araucaria angustifolia* (Bertol.) Kuntze	araucária	0	3	4	LLDP 46
**Bignoniaceae**					
*Tabebuia cassinoides* (Lam.) DC.	caixeta	0	1	2	EAFM 17420
*Handroanthus serratifolius* (Vahl) S.Grose	ipê-amarelo	0	0	1	[36]
**Calophyllaceae**					
*Calophyllum brasiliense* Cambess.	guanandi	4	0	3	FLOR 64032
	guanandi-cedro	1	0	5	FLOR 64033
**Caryocaraceae**					
*Caryocar brasiliense* Cambess.	pequi	1	1	1	[34]
**Euphorbiaceae**					
*Alchornea glandulosa* Poepp. & Endl.	chichá	0	3	0	FLOR 64030
** *Aleurites moluccanus* (L.) Willd.	nogueira	0	2	0	[16]
**Fabaceae**					
*Peltophorum dubium* (Spreng.) Taub.	caubi; caobi	0	0	2	[19]
*Copaifera trapezifolia* Hayne	óleo	0	1	0	[31]
*Senna multijuga* (Rich.) H.S.Irwin & Barneby	angelim	0	0	2	EAFM 17422
* *Hymenolobium* sp.	angelim pedra	0	0	1	[16]
	garacuí, cambará; gracuí	0	1	1	LLDP 15
*Machaerium villosum* Vogel	araribá; ariribá	0	4	0	[32]
*Stryphnodendron* sp.	canafista; canafístula	0	3	3	FLOR 64028
*Anadenanthera colubrina* (Vell.) Brenan	cobi	0	2	0	EAFM 17425
*Schizolobium parahyba* (Vell.) Blake	garapuvu	45	15	97	LLDP 19
	garapuvu-amarelo	0	0	1	[16]
	garapuvu-branco	9	0	15	[16]
	garapuvu-vermelho	9	0	14	[16]
***Tachigali denudata (Vogel) Oliveira-Filho	ingá-amarelo	6	0	11	EAFM 17426
*Tachigali* sp.	ingá-flecha	8	1	7	EAFM 17419
*Hymenaea courbaril* L.	jatobá	0	0	1	[33]
*Enterolobium* sp.	timbaúva	0	1	0	[16]
*Enterolobium contortisiliquum* (Vell.) Morong	timbuva	1	8	7	LLDP 03
	timbuíba-jissara	1	0	1	[19]
****Albizia pedicellaris (DC.) L.Rico	timbuíba-amarela	1	0	0	
	timbuíba-branca	3	0	2	
	timbuíba-rosa	9	1	10	EAFM 17421
	timbuíba	0	0	16	EAFM 17408
*Plathymenia reticulata* Benth.	vinhático	0	1	2	[19]
**Lauraceae**					
*Nectandra* sp. */ Ocotea* sp.	canela	0	23	11	[16]
	canela-garuva	0	2	1	[16]
	canela-preta	0	8	8	LLDP 09; 22
	garuva	0	9	0	[16]
** *Laurus nobilis* Cav.	louro	1	0	3	[19]
**Lecythidaceae**					
*Cariniana legalis* (Mart.) Kuntze	jequitibá	0	2	8	[19, 33]
	jequitibá-rosa	0	0	1	[19, 33]
**Malvaceae**					
*Eriotheca pentaphylla* (Vell. & K.Schum.) A.Robyns	envirussu; embiruçu	0	1	1	[63]
*Pseudobombax* sp.	paineira-imbiruçu	0	1	0	EAFM 17433
*Ceiba pentandra* (L.) Gaertn.	sumaúma	0	1	0	[35]
**Meliaceae**					
*Cedrela fissilis* Vell. / *Cedrela* sp.	cedro	0	37	34	EAFM 17424; 17432; 17418
*Cedrela* sp.	cedro-rosa	0	2	3	[16]
	cedro-vermelho	0	0	1	[16]
**Mimosaceae**					
*Inga* sp.	ingá	0	0	30	[19]
	ingá-cajarana	0	1	4	[19]
**Moraceae**					
*Ficus* sp.	figueira	0	20	8	[16]
	figueira-vermelha	0	0	1	[16]
*Clarisia racemosa* Ruiz & Pav.	oiticica; oiti	0	1	10	LLDP 06
**Ochnaceae**					
*Ouratea* sp.	caquera	0	1	3	EAFM 17423
**Phyllanthaceae**					
*Hieronyma alchorneoides* Allemão	aricurana; urucurana	0	4	2	[33]
**Rubiaceae**					
*Psychotria* sp.	caquera-crespa	1	0	1	EAFM 17429
**Sapindaceae**					
*Cupania vernalis* Cambess.	cambotá	0	1	0	[37]
*Matayba guianensis* Aubl.	ingá-de-concha	0	1	0	EAFM 17417
**Sapotaceae**					
*Manilkara* sp.	maçaranduba	0	3	1	[32]
**Vochysiaceae**					
*Erisma uncinatum* Warm.	cedrinho	0	0	1	[19]
*Vochysia bifalcata* Warm.	guaricica	6	4	3	LLDP 35; 36
Unidentified species		1	18	14	

* The sample of wood cited by the artisan as the name of garacuí (cambará or gracuí) was identified as *Hymenolobium* sp. which is a species known by the name jatobá

** Exotic; species

*** Sclerolobium denudatum ~ Tachigali denudata

**** Balizia pedicellaris ~ Albizia pedicellaris.

The choice of a tree species for a dugout canoe only varied over time among the regions of the Atlantic Forest, which was based on the memory of the artisans (PERMANOVA p [time] = 0.001; p [region] = 0.001). In addition, more species were cited for past than present use (S1 Fig). The analysis of the 358 canoes also confirmed these differences (p [time] = 0.001 gl = 1; p [region] = 0.003 gl [time] = 1; gl [region] = 3).

All of the artisans reported that environmental organs restrict or prohibit cutting trees to construct canoes, mainly cedro (*Cedrela fissilis*), canela (*Nectandra* sp. or *Ocotea* sp., which were very similar for the wood anatomy analysis), peroba (*Aspidosperma* sp.), jequitibá (*Cariniana legalis*) and timbuva (*Enterolobium contortisiliquum*). According to them, environmental monitoring became very strict approximately 20 to 25 years ago. One artisan from Guaraqueçaba (R3) cited there are tree species that no longer exist in the forest, such as arapaçu (Table 1, unidentified species) that was commonly used in the past. Other artisans in R3 (Ariri, Porto Cubatão and Cananéia) also cited this species was used in the past to build dugout canoes. The only canoe of arapaçu analyzed was classified as part of the more recent group, but it was built more than 3 decades ago. The artisans of this region cited ninguvira/nioguvira (unidentified species). According to them, this tree species was commonly used, grows slowly, has noble wood and, therefore, is prohibited to cut.

**Fig 3:**
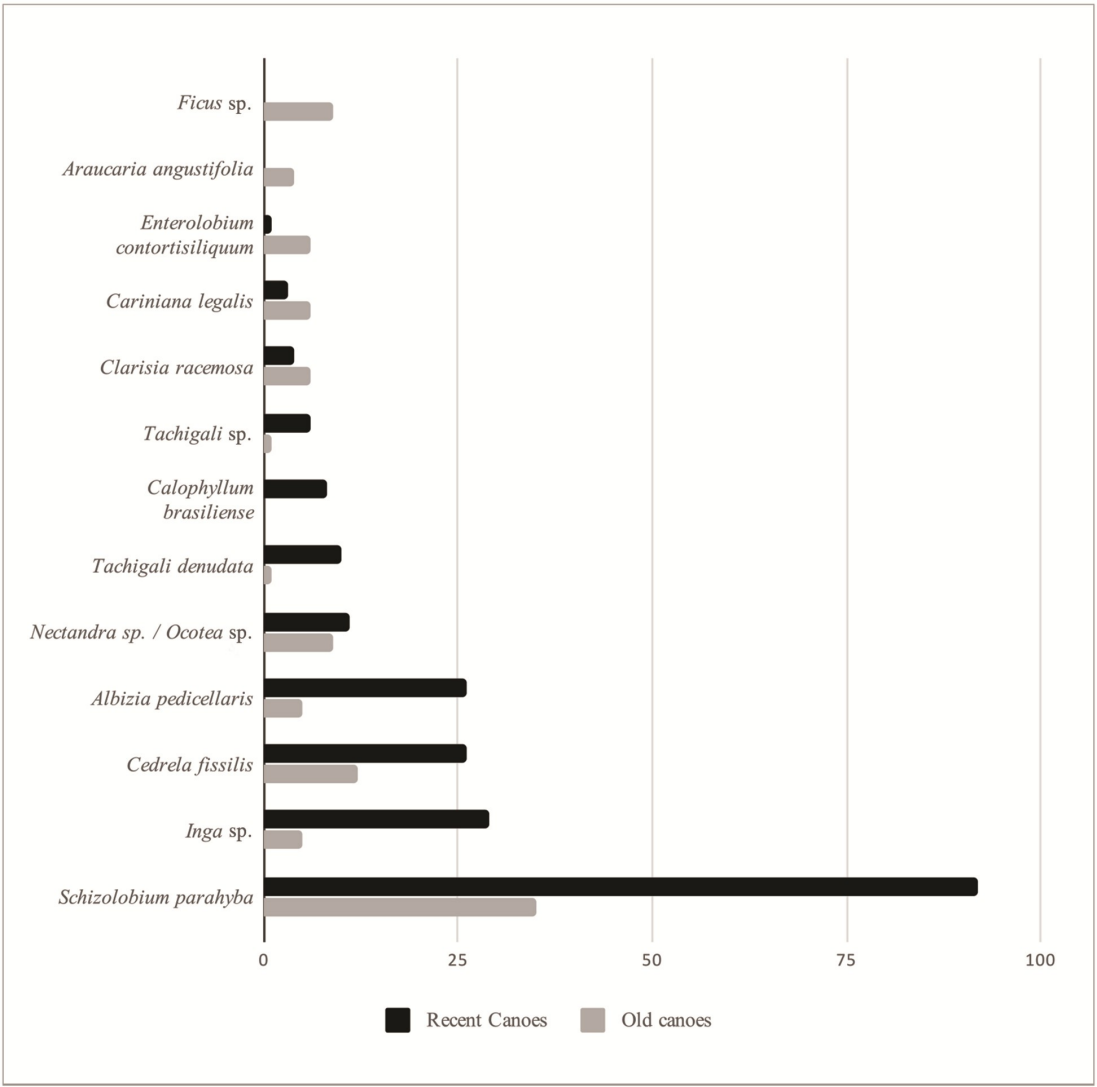
Main tree species used to construct dugout canoes. Canoes over 41 years old—past; up to 41 years old–recent, for species used for at least 4 canoes.

The size of the 358 canoes measured varied over time and older canoes tended to be larger than more recent canoes (F = 119.4; r^2^adj = 0.25; p < 0.01). In the interviews, 62% of the artisans reported there were larger trees in the past (more than 4 decades ago), especially *C*. *fissilis* and *Ocotea* sp. According to the interviewees, the ideal size of the “rodo” (circumference) of the trunk starts at 2 m (equivalent to 63 cm diameter) to make a canoe that is approximately 50 cm wide. Among the most used species in the recent group of canoes (i.e., 250 canoes up to 41 years old), *Schizolobium parahyba*, *Cedrela fissilis/Cedrela* sp., *Albizia pedicellaris*, *Inga* sp. and *Tachigali denudata* predominate; however, among the most used species in the old group (108 canoes), the first two predominate (Fig 3). In addition, the average size of the old canoes made of *C*. *fissilis* differs significantly from the recent canoes made of this species (old average = 0.9 m sd = 0.14; recent average = 0.67 m sd = 0,1; t = 5.467; p<0.01). The same occurs for *S*. *parahyba*; the more recent canoes (average = 0.71 sd = 0,14) are smaller than the older canoes (average = 0.94 sd = 0,17) (t = 7.5675; p<0.01). Some of the tree species cited by the artisans as only used in the past, for example, figueira (*Ficus* sp.), araucária (*Araucaria angustifolia*) and timbuva (*Enterolobium contortisiliquum*), were also identified only among the old canoes, which corroborates the data obtained from the interviews.

**Fig 4:**
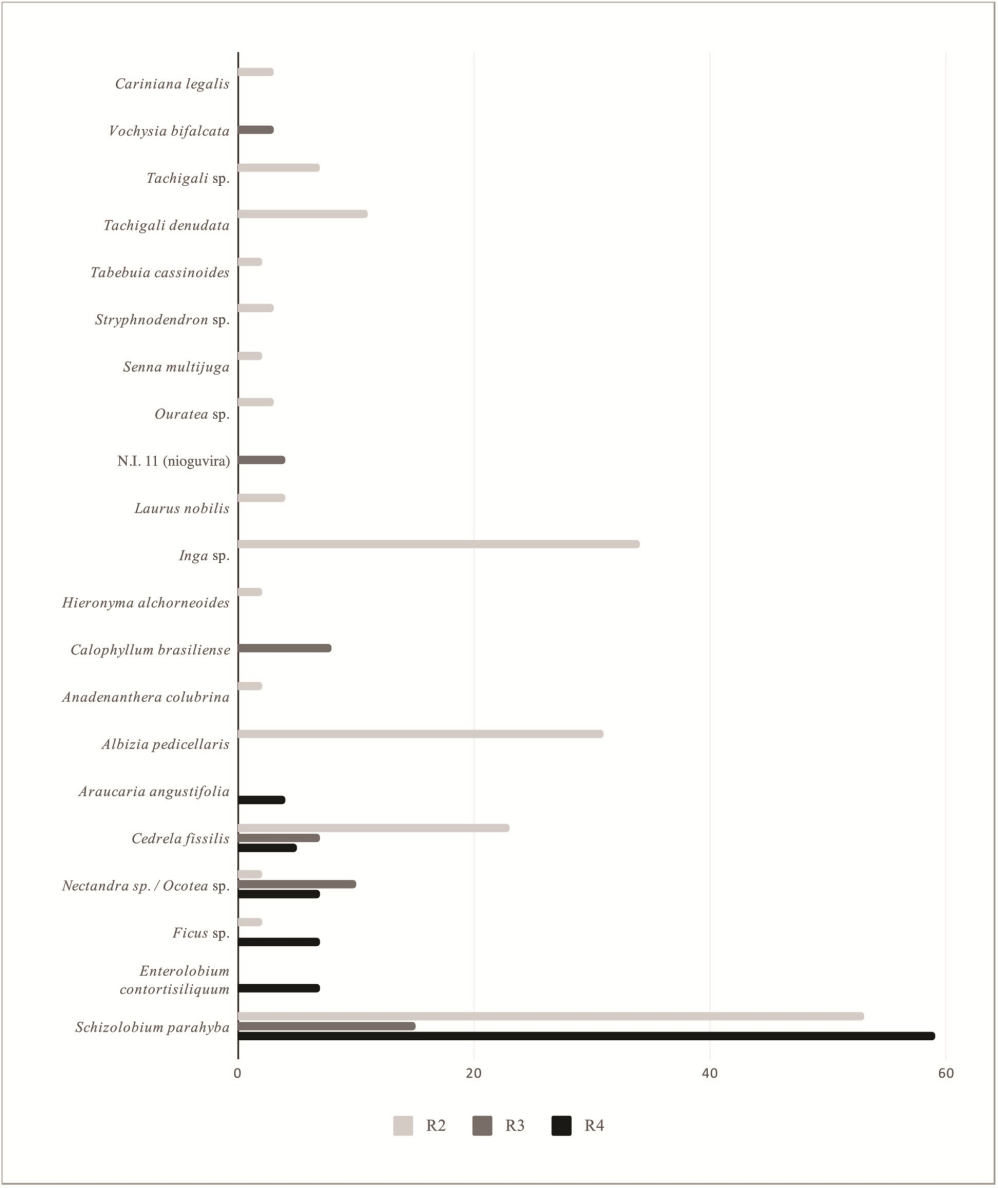
Tree species used to construct dugout canoes per region. N.I. = unidentified, R2 = Paraty / Ubatuba, R3 = Lagamar, R4 = Coast of Santa Catarina for species used for at least 2 canoes.

The main tree species used to build dugout canoes also varied among the regions (Fig 4). Regions R2, R3 and R4 had only *S*. *parahyba*, *C*. *fissilis* and *Nectandra* sp./*Ocotea* sp. in common. Region 1 was not included in this analysis because canoes in this region are constructed with wood from other areas, mainly the states of Bahia and Espírito Santo. Canoes in R1 are usually made from *Clarisia racemosa*, as well as *Plathymenia reticulata*, *Handroanthus serratifolius* and *Cariniana legalis*. Artisans in R2 reported a mortality event of *S*. *parahyba* in Saco do Mamanguá, Ponta Negra and Praia do Sono. Some of them associated this event with the aging of the trees or a disease caused by a fungus in the roots, making this wood less available during recent times.

**Fig 5:**
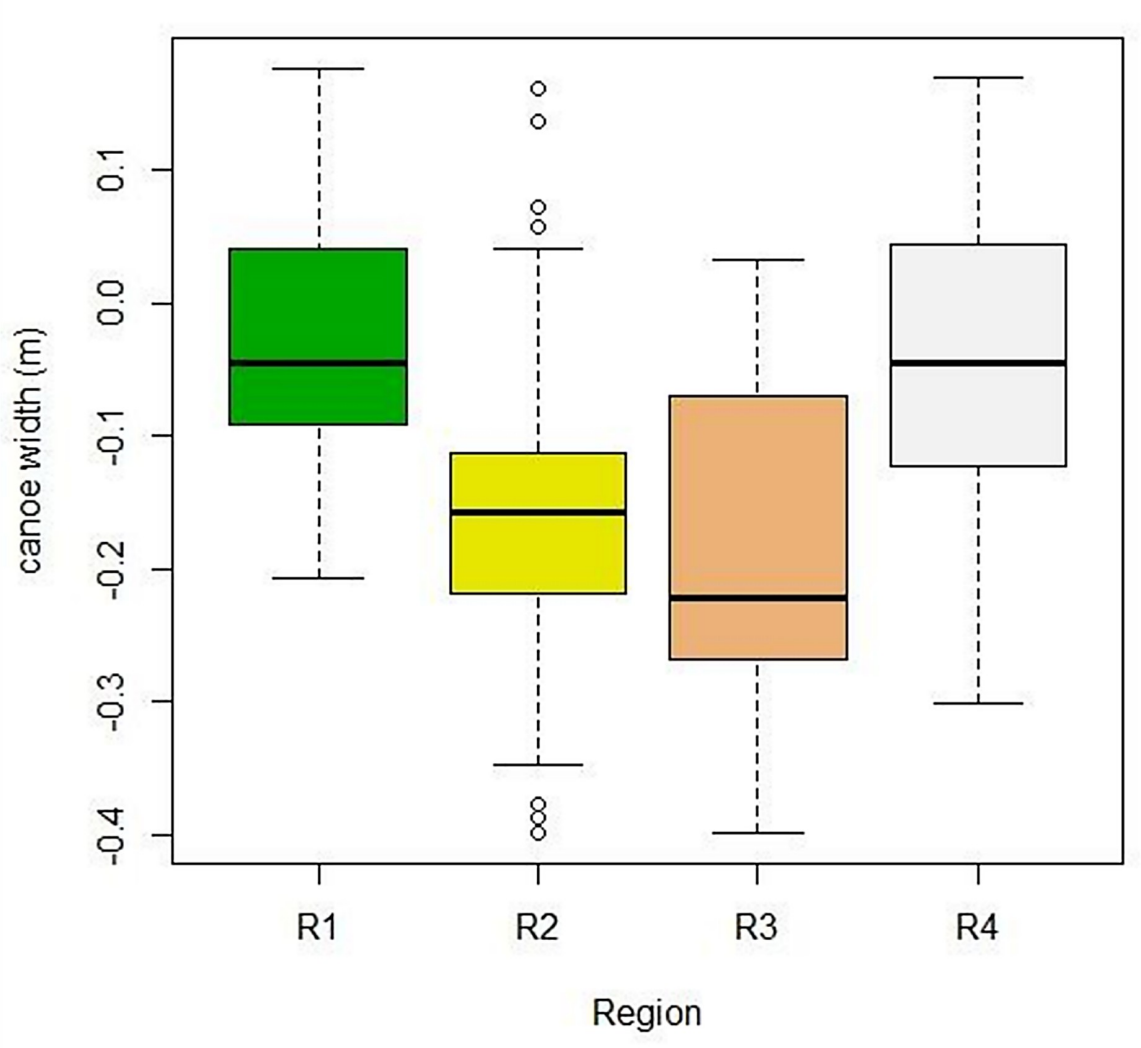
Width (m) of 358 canoes measured in four regions along the southern and southeastern Brazilian coast. R1 = Cabo Frio Region, R2 = Paraty / Ubatuba, R3 = Lagamar, R4 = Coast of Santa Catarina.

Canoe size varied between regions (f = 44.67; gl = 3; p<0.01): between R1 and R3, R4 and R3, R1 and R2 and R4 and R2 (Fig 5). Regions 1 and 4 have an average width of 0.9 m (sd = 0.15 and 0.2, respectively), and regions 2 and 3 have an average width of 0.7 m (sd = 0.15) and 0.6 m (sd = 0.18), respectively.

The size of the canoes used in sheltered and exposed environments differs significantly (Wilcoxon W = 4396.5; p<0.01). The average width of the canoes was 0.7 m (sd = 0.15) for the sheltered environment and 0.97 m (sd = 0.22) for the exposed environment. The average width of the canoes in R4 was considerably greater than that of R2 and R3. In R4, 55% of the canoes are used in open ocean along beaches that generally have larger waves were canoes need to pass the surf. In R3, 73% of the canoes are used in sheltered environments. In R2, 89% of the canoes are used in coves, bays and along more protected beaches.

The main type of fishing was with a gillnet (163 canoes), followed by a purse seine net (155 canoes) and fishing with a line and hook (134 canoes). Other types identified were nets to catch mullet (tainha), fishing with a cast net (tarrafa), fyke net (covo) and dragnet (gerival), as well some methods that are less common. There were differences between canoe size and type of fishing exercised (kw = 277.99; p<0.01; gl = 13). The methods that use the larger canoes were fishing with a trawl net and a net for mullet (8.28 m; sd = 0.9 and 8.11 m; sd = 1.02 respectively), and those that use the smallest canoes were fishing with a gillnet (4.85 m; sd = 0.9), purse seine net (5.02 m; sd = 1.42) and with a line and hook (4.85 m; sd = 0.9). According to the artisans, the dimensions of the canoe vary based on the amount of weight carried during fishing, which is related to the number of people in the canoe, the fishing gear and the type of fishing, as well as the environment were the canoe is used.

In some coastal points, mainly in areas of R3 and R1, we observed the intense substitution of dugout canoes with fiberglass canoes and motor boats. Nearly 100% of the canoes encountered in Pontal do Paraná, Pontal do Sul, Matinhos and Guaratuba (R3) were fiberglass an identical to dugout canoes. In Iguape, Icapara and Ilha Comprida (R3) we also encountered many fiberglass canoes identical to dugout canoes. Fishers and artisans of this region reported that fiberglass canoes play the same role as wooden canoes but are easier to maintain, acquire and build, especially because authorization to cut a tree is not needed. Fishers in R1 reported that after environmental monitoring increased, the practice of sending wooden canoes to the region became risky, resulting in the use of other boat types, such motorized aluminum boats (voadeiras). In contrast, on Santa Catarina Island (R4) dugout canoes are considered works of art and are very well cared for by their owners, which is mainly because they are used in artisanal fishing for mullet, which is still widely practiced and part of the local cultural identity.

## Discussion

The artisans of southern and southeastern Brazil had different preferences over time in relation to the tree species chosen to make the hull of dugout canoes. The most cited species for current use were garapuvu (*Schizolobium parahyba)*, timbuíba rosa (*Albizia pedicellaris)*, ingá flecha (*Tachigali* sp.) and ingá amarelo (*Tachigali denudata*), which have also been described by other authors [19, 40, 41]. More than half of the artisans mentioned that species are easy to find in the forest, mainly *S*. *parahyba* in R3 and R4 and *A*. *pedicellaris* in R2, reaffirming recent studies [11, 42, 43] that describe these species as abundant trees in the forest.

*Schizolobium parahyba* is a pioneer species [44, 45] that quickly grows [46] and easily germinates [47]. It occurs in hillside forests in the South and Southeast regions of Brazil [47, 48] and reaches and adequate size for building canoes [11]. All of the artisans in R4 cited *S*. *parahyba* as the most used tree to build dugout canoes, which was also observed by our colleagues [11, 16]. According to Orofino et al. (2017) [11], itinerant farming activities practiced in the past (until around 1970) on Santa Catarina Island, combined with sporadic extraction of trees for dugout canoes, contributed to an increase in the availability of *S*. *parahyba* in this region. The artisans consider *S*. *parahyba* to have a straight trunk with wood that is resistant to salt water and easy to work (light and easy to cut). Its use predominates in the past and present. Of the 358 canoes analyzed, 127 were made of this species, and this species was the most used for canoes in R2, R3 and R4. According to historical records from the XVIII century, in a list of trees that produce useful wood on Santa Catarina Island, *S*. *parahyba* is cited as a tree used to construct high-quality canoes that were in vogue at the time [32], revealing that the availability and use of this tree species are not recent.

*Albizia pedicellaris* (= *Balizia pedicellaris*; [30]) is commonly used to build dugout canoes in the region of Paraty (R2), due to its large size and vast occurrence, and it is easy to find specimens of this species that can still be used [49]. The species is widely distributed and occupies the Amazon, Cerrado and Atlantic Forest phytogeographic domains, from Amazonas to Maranhão, down through the central plateau, until the Atlantic coast of Paraná [50]. Lorenzi (2002) [29] notes that this species prefers the interior of primary forests and “capoeirões” (old successional forest) on hillsides.

*Tachigali denudata* (= *Sclerolobium denudatum*; [30]), popularly known as ingá amarelo, is endemic to the Atlantic Forest in the states of Paraná, São Paulo and Rio de Janeiro in the Southeast Region of Brazil [51]. According to Cassetari (2010) and Campos et al. (2011) [42, 43], it is abundant and frequent in northeastern São Paulo, which could be why it is one of the most frequent species in R2 (Paraty, in southern Rio de Janeiro and Ubatuba, São Paulo).

Canela (*Ocotea* sp.; *Nectandra* sp.), cedro (*Cedrela fissilis)* and figueira (*Ficus* sp.) were cited by the artisans as the most used species in the past. Other authors report the use of these woods to construct dugout canoes [1, 11, 47, 52]. Câmara (1937) [6] in the section “Madeiras de construção” (Woods for construction), cited canela (*Nectandra* sp.), bacurubú (*Schizolobium robustum*, now *Schizolobium parahyba*), cedro (*Cedrela* sp.), figueira brava (*Ficus doliaria*) and peroba (*Aspidosperma peroba*) as woods used to construct canoe hulls, reaffirming they were species used in the past. Like the present study, canoe builders in Ilhabela evaluated figueira as a wood of poor durability [40]. Caruso (1990) and Várzea (1984) [32, 53] also cited the use of figueira (*Ficus* sp.) to construct dugout canoes, corroborating that the species was used in the past. As observed in the present study, canela was considered a good quality wood for constructing canoe hulls [11, 36], in addition to being heavy and better adapted to freshwater use [11].

*Ocotea* sp. and *C*. *fissilis* were considered woods of good quality and workability, with ideal trunks for making canoes, but presently less available. Among the species in Brazil threatened with extinction in the Atlantic Forest biome are some that have been extensively exploited for various purposes, including species of canela (canela-preta, *Ocotea catharinensis*; canela-sassafrás, *Ocotea odorifera*) and cedro (*Cedrela fissilis*) [25]. In the XVIII century, the “madeiras de lei” (hardwoods) on the Island of Santa Catarina, such as *Ocotea catharinensis*, *Cedrela fissilis*, maçaranduba (*Manilkara subsericea)*, óleo (*Copaifera trapezifolia*) and peroba (*Aspidosperma pyrifolium*), were used for civil construction, furniture, canoes and whaling boats, masts and parts for ships, and were also exported [32]. In association with this partial and selective deforestation, pressures from extracting wood for energy and opening areas for cultivation contributed to deforestation until the middle of the XX century; starting then, the vegetation was reestablished on abandoned agricultural land and by 1978 55% of the island was covered by secondary vegetation at different stages of succession [32]. More than half of the artisans noted that around 40 years ago there were very large trees of (especially cedro and canela) that were ideal for building canoes and that presently these are rare.

The average size of the old canoes of *C*. *fissilis* was significantly greater than the recent canoes made with this species. This reveals that larger individuals were used over 40 years ago compared to more recently, suggesting that larger individuals of this tree existed in the past compared to those currently available. The most robust individuals, with the greatest diameters, were the most exploited in the Atlantic Forest [54, 55], which is reflected in the size of the individuals found today, mainly for species of slow-growing hardwoods, such as *C*. *fissilis*.

The ideal diameter of a tree for a canoe reported by the artisans was 60 cm, which is close to what the other study [11] described. An inventory of forest remnants in Santa Catarina recorded DBH averages of 23 ± 1.65 cm for *C*. *fissilis*, 24.27 ± 2.08 cm for *O*. *catharinensis*, and 26.85 ± 12.14 cm for *Ficus* sp. [56]. These dimeters are smaller than the ideal diameter indicated by the artisans. The difference between the averages reported by the artisans and those reported by this study [56] might be due the existence of few large individuals of the species cited, since they are presently rare in the forest. Orofino (2017) [37] reported that the DBH average of individuals of *S*. *parahyba* was 55.36 ± 13.93 cm in areas that were under crop rotation and had been fallow for at least 60 years, indicating that historically managed locations can be sources of trees with more appropriate dimensions for canoe building.

We encountered 24 canoes made of *C*. *fissilis* that were less than 40 years old, contradicting the perception of the artisans that this species was used only in the past. Since cutting this species is prohibited or restricted, the artisans could have been hesitant to report that it is still used, even though they were using smaller individuals. Most artisans cited that environmental monitoring became more stringent around 20 years ago. In 1998, the first federal law (nº 9605/1998) was created that effectively penalized people for harming the environment [57] which is the same time period that the artisans cited for when environmental organs increased the prohibition or restriction of cutting trees, mainly those considered hardwoods (“*madeiras de lei*” or “*madeiras nobres*”).

Of the 20 analyzed canoes made of *Ocotea* sp./*Nectandra* sp., only five were constructed two decades ago. The rest are estimated to be three or more decades old, including two estimated to be a century old and another estimated to be two centuries old. Canela-preta (*Ocotea* sp.) was the species of canela most cited by the artisans, especially in R3 and R4. It was the most common and characteristic tree of dense ombrophilous forest in Santa Catarina (R4), representing 1/3 of the volume of all wood per hectare [44], but is now a species threatened with extinction [58] due to being heavily exploited [59].

The species *Enterolobium contortisiliquum* and *Araucaria angustifolia* were identified predominantly for the group of old canoes (more than 41 years old) and only in R4. The artisans reported these species are low in availability, slow growing and could not be cut. The individuals of *A*. *angustifolia* were probably from mixed ombrophilous forest at least 100 km from the coast and this tree is on the list of species threatened with extinction [58].

Along the coast of the study area there are differences in relation to the availability of trees for canoes. The northern part of R1 corresponds to the Cabo Frio Center of Plant Diversity [60, 61], where the construction of dugout canoes was never practiced due to the absence of large trees; although, these boats are still used in the open ocean and lagoon environments. This region is in the Atlantic Forest domain but has geomorphological and climatic peculiarities [62] that are reflected in its physical and biological heterogeneity, such as plant diversity (physiognomic and floristic) and a high degree of endemism, which is probably associated to its paleo-evolutionary history, remaining remnants of vegetation form the Pleistocene glacial periods, with xerophytic formations along the coast, and arboreal stratum with trees that do not become tall due to the predominance of winds from the north [61].

Dugout canoes are built in R2, especially with *Cedrela fissilis* and *Schizolobium parahyba*, and R2 is the only region that uses *Albizia pedicellaris* and *Tachigali denudata* (also reported by [36] and [63] for the region of Paraty). Although there are records of using *S*. *parahyba* to construct canoes in the region of Paraty, at the end of the XIX century [6], currently using this species is rare. The mortality of this species reported by the artisans interviewed in this study was discussed by Callado and Guimarães (2010) [64], who cited atypical annual precipitation between the years of 1997 and 2001 in the region as the cause of death.

In the Lagamar complex (R3), the woods most cited by the artisans for recent use were *Schizolobium parahyba*, *Vochysia bifalcata*, and *Calophyllum brasiliense*. In R4, the use of *S*. *parahyba* was cited the most for both the present and the past. Historical records from the XVIII century [65] note the use of a set of species similar to what was found in the present study, including figueira (*Ficus* sp.) and canela (*Ocotea* sp. */ Nectandra* sp). In 1900, “Figueira brava” (*Ficus doliaria*) and “Guapurubu” (*Schizolobium excelsum*, synonym of *S*. *parahyba* [66]) were considered the most used [53].

The variety of tree species used to construct canoes in each region is reflected in the structure and composition of species in coastal Atlantic Forest, which is very diverse and differs based on location [67]. The successional stages of coastal forests in this biome exhibit similar patterns, but the composition of these forests is highly variable and depends on, for example, latitude [67–69] and intensity of perturbation in the environment [67]. Due to the previous use of areas of Atlantic Forest, mainly for subsistence agriculture, many present remnants are secondary forest fragments under various pressures [70], which has resulted in the transformation of the landscape in different regions of the biome over time.

We observed that there was significant variation in canoe size based on the region (R1, R2, R3, R4) and environment (sheltered or exposed) where the canoe is used. Region and environment of use can be associated variables, since the four studied regions along the southern and southeastern coast have diverse landscapes, from more exposed environments with different types of beaches to more sheltered environments, such as estuaries composed of canals and rivers in R3 [20–22]. Rodrigues (2005) [71] also discussed the size of canoes and environment of use, where a smaller size was used for navigating rivers and a larger size was used in the ocean.

Variation in canoe size is also related to the type of fishing. The average canoe size used to fish for mullet or with a trawl net is larger (8m long) than the average size used to fish with a line and hook or throw net (5 to 6m long). In Ilhabela (R3), canoes of 3 to 4 m are used for coastal fishing, using a line technique to fish for squid, those of 4 to 7 m are used for techniques called fixed pound net (cerco fixo) and floating pound net (cerco flutuante) and/or to fish with other types of nets, and those over 7 m are used to fish with a net or for transport [40]. Similarly, in the central region of Santa Catarina (R4) the smaller canoes (< 4 m and between 4 to 8 m long) are currently used more because they are easier to maneuver and it is possible to fish alone or with a small crew; however, during mullet season (May to July) larger canoes are used because the nets for this type of fishing are larger, more fish are caught and the boats need to pass through the surf [11].

Thus, we noticed that the variation in canoe size is related not only to canoe age (which refers to the temporal scale and use of larger trees in the past), but also to the region, environment, and type of fishing. This can explain the low r value in the linear regression for canoe size in function of age, where age explains only 25% of the variation in size.

Although dugout canoe building is in decline, especially due to more environmental monitoring that has increased the bureaucracy to obtain permission to cut trees, and because the canoes are difficult to maintain compared to other boats, there are regions where the tradition is still practiced, for example, Saco do Mamanguá (SP), Ariri (SP), Antonina (PR), and Florianópolis (SC). On Santa Catarina Island, even though building new canoes is currently sporadic, these boats still play an essential role in daily activities, such as transporting people and products [11] and artisanal fishing, mainly for mullet that is done with dugout gunwale canoes (canoas bordadas). Fishers that still use dugout canoes mainly from the regions cited above, want to maintain the traditional fishing activity, which is fundamental to sustaining their way of life [41, 72].

## Conclusion

The differences encountered for the availability of tree species and size of individual trees over time are possibly related to changes in the vegetation of the Atlantic Forest biome over time that, from a historical perspective, are a result of how past and recent populations interacted with the environment. The forests we have today are systems that have been managed for centuries and are in constant transformation. In addition to the changes in the vegetation, latitudinal variation among the regions can also influence the availability of species in the regions, since there is local variation in the structure and composition of species diversity. Although the canoes are constructed from a single tree trunk and the results indicate that trees used in the past were larger than those recently or currently used, from a temporal perspective, it was not possible to find a simple relation between canoe size and tree used, since the canoes not only vary in relation to time, but also in relation to region, environment, and type of fishing. We verified the influence of elements that contributed to the decline in dugout canoe building, including an increase in environmental monitoring of cutting trees, cheaper maintenance, and ease of building fiberglass canoes. This favored changes in the habits of traditional populations, resulting in the substitution of dugout canoes for fiberglass canoes and motor boats.

Some of the tree species used to construct canoes are associated with wood that has a widespread history of use, both in the past and present. Today, some of the largest and oldest canoes are examples of large trees that existed in the studied regions, compared to those recorded in recent inventories, and represent real historical fragments of the Atlantic Forest. Thus, the ethnobotanical study of dugout canoes can be used as a tool to reveal pieces of history of this important biome.

## Supporting information

S1 Fig Relative frequency of the tree species used for the construction of the dugout canoe, according to 53 artisans, in the present and in the past. N.I. = unidentified.
(TIF)

S1 File Questionnaire used in the interviews with artisans. (DOCX)

S1 Table Interview and dugout canoes were found along the four regions under study. (DOCX)
