# Supplementary figures and images for: Artisans and dugout canoes reveal pieces of Atlantic Forest history
Source: PLoS One. 2019 Jun 26;14(6):e0219100. doi: 10.1371/journal.pone.0219100 (PMC6594645; doi:10.1371/journal.pone.0219100)

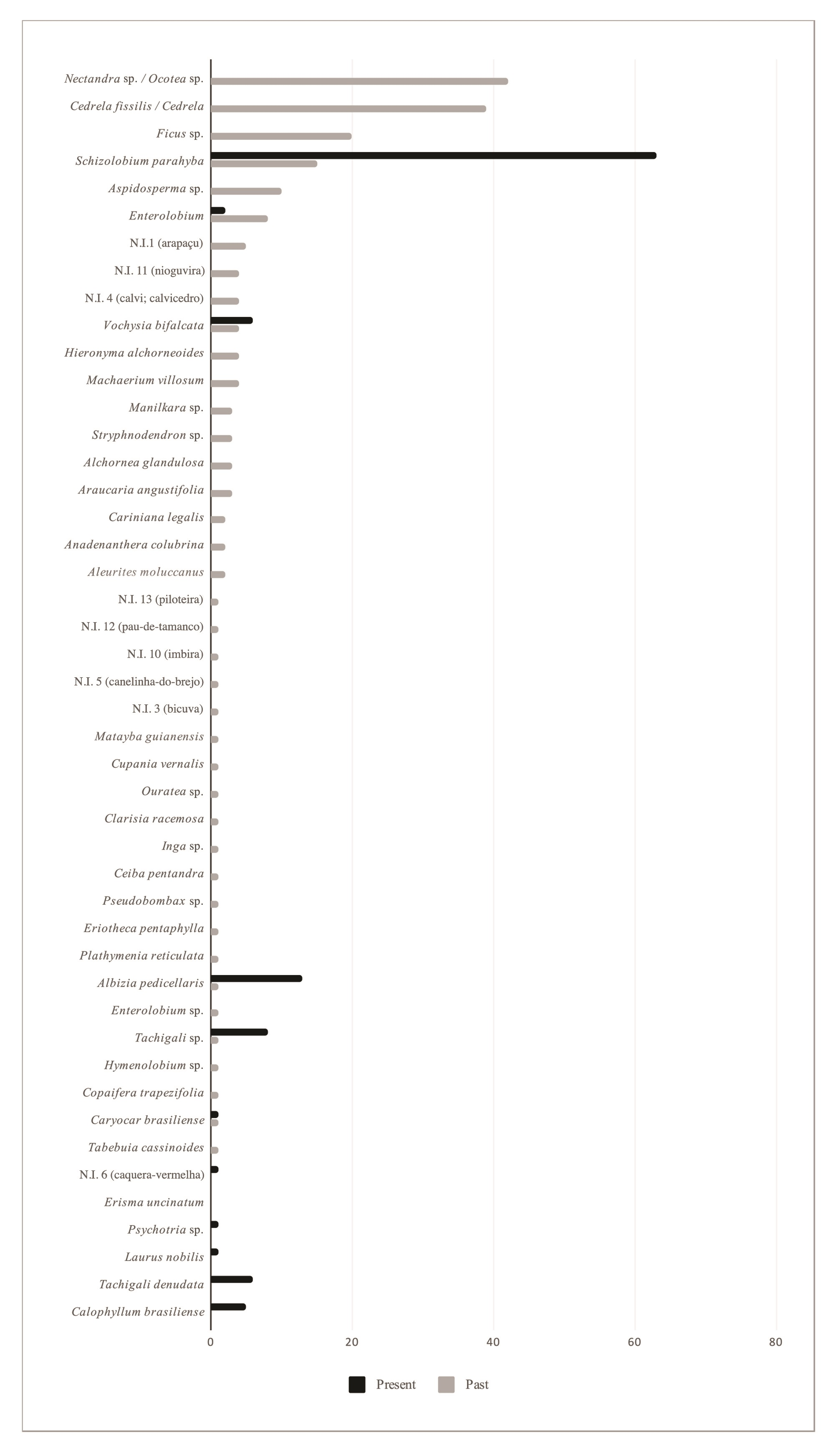

Supplement: S1 Fig — N.I. = unidentified. (TIF) [file pone.0219100.s001.tif]
