# Supplementary material for: Artisans and dugout canoes reveal pieces of Atlantic Forest history
Source: PLoS One. 2019 Jun 26;14(6):e0219100. doi: 10.1371/journal.pone.0219100 (PMC6594645; doi:10.1371/journal.pone.0219100)
Supplement: S1 File — (DOCX) [file pone.0219100.s002.docx]

**Questionnaire used in the interviews with artisans**

Number of the interview: Interviewer:

Date:

Municipality:

Locality:

Name of the interviewee: Gender:

Age: Profession/Economic activities:

1. Which is the most used tree to make canoes currently? Why?
2. Which is the most used tree to make canoes In the past (when?) were there other trees used to make canoes? If yes, why they are not currently used?
3. Were there any larger trees (what size?) than they are nowadays? If yes, when?
4. There were more trees to make canoes in the past than nowadays? Why?
5. Are the oldest canoes (from when) bigger (estimate size)? Why?
6. What kind of canoe is best for fishing here? Which kind of fisheries are practiced here?
7. In which environment(s) the canoe is used?
