# Supplementary material for: Artisans and dugout canoes reveal pieces of Atlantic Forest history
Source: PLoS One. 2019 Jun 26;14(6):e0219100. doi: 10.1371/journal.pone.0219100 (PMC6594645; doi:10.1371/journal.pone.0219100)
Supplement: S1 Table — (DOCX) [file pone.0219100.s003.docx]

**S1 Table**. Division of the four coastal regions of the crystalline cliffs: interview sites and / or sites visited and / or where dugout canoes were found along the south-southeast Brazilian coast.

| **Region** | **Region name** | **Brazilian State** | **Sites** | **Latitude** | **Number of Interviews** | **Number of Canoes** |
| --- | --- | --- | --- | --- | --- | --- |
|  |  |  |  |  |  |  |
| R1 | Cabo Frio to Rio de Janeiro | Rio de Janeiro | Arraial do Cabo, Araruama, São Pedro da Aldeia, Niteroi, Barra de São João | 22°S | 1 | 26 |
| R2 | Paraty and Ubatuba | Rio de Janeiro and São Paulo | Ubatuba, Paraty (Praia do Sono, Saco do Mamanguá, Ponta Negra, Cairuçu das Pedras), Ilha Grande | 23°S | 9 | 192 |
| R3 | Lagamar | São Paulo and Paraná | Iguape (Barra do Ribeira, Icapara, Iguape), Ilha Comprida, Cananeia (Pedrinhas, São Paulo Bagre, Cananeia, Porto Cubatão, Ariri), Antonina, Pontal do Sul, Pontal do Paraná, Guaraqueçaba, Paranaguá, Ilha dos Valadares, Matinhos, Guaratuba. | 24° S and 25°S | 17 | 49 |
| R4 | Costa de Santa Catarina | Santa Catarina | Florianópolis, Governador Celso Ramos, Palhoça, Bombinhas, Bombas, Imbituba, Garopaba, Laguna. | 27° S and 28°S | 26 | 91 |
